# Stemness Characteristics of Periodontal Ligament Stem Cells from Donors and Multiple Sclerosis Patients: A Comparative Study

**DOI:** 10.1155/2017/1606125

**Published:** 2017-12-14

**Authors:** Francesca Diomede, Thangavelu Soundara Rajan, Marco D'Aurora, Placido Bramanti, Ilaria Merciaro, Marco Marchisio, Valentina Gatta, Emanuela Mazzon, Oriana Trubiani

**Affiliations:** ^1^Stem Cells and Regenerative Medicine Laboratory, Department of Medical, Oral and Biotechnological Sciences, Università “G. d'Annunzio”, Chieti-Pescara, Via dei Vestini 31, 66100 Chieti, Italy; ^2^IRCCS Centro Neurolesi “Bonino-Pulejo”, Via Provinciale Palermo, Contrada Casazza, 98124 Messina, Italy; ^3^Department of Psychological, Health and Territorial Sciences, School of Medicine, Università “G. d'Annunzio”, Chieti-Pescara, Via dei Vestini 31, 66100 Chieti, Italy; ^4^Department of Medicine and Aging Science, Università “G. d'Annunzio”, Chieti-Pescara, Via dei Vestini 31, 66100 Chieti, Italy

## Abstract

Multiple sclerosis (MS) is the most prevalent and progressive autoimmune disease that affects the central nervous system, and currently, no drug is available for the treatment. Stem cell therapy has received substantial attention in MS treatment. Recently, we demonstrated the immunosuppressive effects of mesenchymal stem cells derived from neural crest-originated human periodontal ligament tissue (hPDLSCs) in an *in vivo* model of MS. In the present study, we comparatively investigated the stemness properties of hPDLSCs derived from healthy donors and relapsing-remitting MS patients. Stem cell marker expression, cell proliferation, and differentiation capacity were studied. We found that both donor- and MS patient-derived hPDLSCs at early passage 2 showed similar expression of surface antigen markers and cell proliferation rate. Significant level of osteogenic, adipogenic, chondrogenic, and neurogenic differentiation capacities was observed in both donor- and MS patient-derived hPDLSCs. Interestingly, these cells maintained the stemness properties even at late passage 15. Senescence markers p16 and p21 expression was considerably enhanced in passage 15. Our results propose that hPDLSCs may serve as simple and potential autologous stem cell niche, which may help in personalized stem cell therapy for MS patients.

## 1. Introduction

Multiple sclerosis (MS) is a chronic debilitating neuroinflammatory disease, which resulted from the activation of immune response against self-antigens residing in the central nervous system (CNS). Activated immune cell infiltration in the brain and spinal cord, degenerated myelin sheath, and severe axonal damage are the typical pathological signatures of MS, which eventually cause severe neurological disabilities [1, 2]. Relapsing-remitting-MS (RR-MS) is the most common form of MS with 85% incidence rate, and there is no cure till date [3]. Accordingly, developing new therapeutics for this dreadful disease is very urgent. Mesenchymal stem cells (MSCs), owing to their tissue regenerative and immunomodulatory characteristics [4], have become the center of attraction for MS treatment, and a considerable amount of stem cell-based clinical trials has been made so far with promising results [5]. MSCs are adult stem cells present in tissues including dental, adipose, bone marrow, and placenta. MSCs are renowned for their self-renewal capacity and differentiation efficacy towards various kinds of cells such as adipocytes, osteocytes, myocytes, and neurons [6].

In recent years, neural crest-originated nonhematopoietic MSCs derived from human dental tissues have attained substantial attention in the field of regenerative medicine for dental and nondental diseases [7]. Human dental MSCs are obtained from various types of dental tissues such as periodontal ligament, dental pulp, and gingiva [8]. MSC isolation from adult tissues require invasive procedures such as liposuction for adipose-derived MSCs and aspiration for bone marrow-derived MSCs [9]. Conversely, a minimal surgical procedure is required for dental tissue-derived MSCs, suggesting the possibility of dental tissues to serve as simple and compelling autologous stem cell resources for stem cell-based therapy in MS patients.

In the present study, we investigated whether human PDLSCs (hPDLSCs) could serve as an effective autologous tool for RR-MS patients. To this end, we studied the stemness characteristics of hPDLSCs derived from RR-MS patients in comparison to those of healthy subjects. Cell surface antigen expression, cell proliferation rate, and differentiation capacity were examined. In addition, we investigated the putative modulation of stem cell properties after prolonged *in vitro* cultures using hPDLSCs at early (2nd) and late (15th) passages.

## 2. Materials and Methods

### 2.1. Ethic Statement

To perform this study, the authors obtained an approval statement from the Ethics Committee at the Medical School, Università degli Studi G. d'Annunzio Chieti-Pescara, Italy (n°266/17.04.14). Informed consent was signed by all patients before sample collection.

### 2.2. Cell Culture Establishment

Human PDLSCs were isolated from periodontal tissues of healthy donors (*n* = 3) and RR-MS patients (*n* = 3) as previously described by Rajan et al. [10]. Cells were cultured in MSCGM-CD medium (mesenchymal stem cell growth medium-chemically defined) (Lonza, Basel, Switzerland) and were incubated at 37°C in a humidified atmosphere of 5% CO_2_ in air. Human PDLSCs from the second (P2) and the fifteenth passages (P15) were used for the study.

### 2.3. Flow Cytometry

#### 2.3.1. Antibodies

Fluorescein isothiocyanate- (FITC-) conjugated CD14, phycoerythrin- (PE-) conjugated CD29, CD31-FITC, peridinin chlorophyll protein- (PerCP-) cyanine (Cy)5.5-conjugated CD34, CD44-FITC, CD45-FITC, CD73-PE, CD90-FITC, CD105-PE, CD166-FITC, CD326-PerCP-Cy5.5, Alexa488-conjugated human leukocyte antigen- (HLA-) ABC, and HLA DR-PE were used (BD Biosciences, Franklin Lakes, NJ; https://www.bdbiosciences.com). Pluripotent embryonic markers NANOG/PE, octamer-binding transcription factor 3/4 (Oct3/4)/PE, stage-specific embryonic antigen (SSEA4)/FITC, and sex-determining region Y-box 2 (SOX2)/FITC were purchased from BD Biosciences.

#### 2.3.2. Cell Staining

Samples were stained for surface or intracellular antigens, as previously described by Rajan et al. [10]. Cells were analyzed on a FACSCanto II flow cytometer (BD), using DIVA™ software (Becton Dickinson).

#### 2.3.3. Flow Cytometry Measurement

Quality control included regular check-up with Rainbow Calibration Particles (6 peaks) and CaliBRITE beads (both from Becton Dickinson). Debris was excluded from the analysis by gating on morphological parameters; 20,000 nondebris events in the morphological gate were recorded for each sample. All antibodies were titrated under assay conditions, and optimal photomultiplier (PMT) gains were established for each channel.

### 2.4. Cell Viability Assay

MTT assay and trypan blue exclusion test were deployed to evaluate cell proliferation and viability at different time points (24 h, 48 h, 72 h, and 1 week) as previously described by Rajan et al. [11]. As regards trypan blue exclusion test, cells were counted at selected time points using an algorithm available online (http://www.doubling-time.com).

### 2.5. Induction of Mesengenic Differentiation

Human PDLSCs and MS-hPDLSCs at P2 and P15 were subjected to mesengenic differentiation as reported by Rajan et al. [12]. Cells were stained with Alizarin Red S, Adipo Oil Red, and Alcian blue solutions in order to analyze osteogenic, adipogenic, and chondrogenic differentiation, respectively. Images were captured using inverted light microscopy (Leica DMIL, Leica Microsystem, Milan, Italy).

### 2.6. Induction of Neurogenic Differentiation

For neurogenic differentiation, hPDLSCs and MS-hPDLSCs were plated in 24-well plates and were induced with Neurobasal-A Medium (Gibco®) containing B27 (2%), L-glutamine (2 mM), penicillin (100 U/ml), streptomycin (100 mg/ml), and amphotericin B (5 mg/ml) (neuroinductive medium) and supplemented with basic fibroblast growth factor (bFGF, 20 ng/ml) (Tema Ricerca, Milan, Italy) for 10 days [13]. To evaluate the differentiation, cells were processed for *β*III-tubulin immunostaining detection and were observed using confocal laser scanning microscopy (LSM5META, Zeiss, Jena, Germany).

### 2.7. *β*-Galactosidase Staining

Cells were stained using a senescence detection kit (Ab65351, Abcam, Cambridge, UK) as per the manufacturer's instructions. The percentage of positively stained cells (blue cells) versus total cells was calculated by randomly choosing 10 microscopic fields under 10x objective magnification at light microscopy (Leica DMIL, Leica Microsystem).

### 2.8. Immunofluorescence

hPDLSCs and MS-hPDLSCs were processed as previously reported by Trubiani et al. [13]. Cells were incubated first with primary monoclonal antibodies: anti human p16 (1 : 200, rabbit) (Santa Cruz Biotechnology Inc., Santa Cruz, CA, USA) and p21 (1 : 50, rabbit) (Santa Cruz Biotechnology). Then cells were incubated with secondary antibody: Alexa Fluor 568 red fluorescence-conjugated goat anti-rabbit (1 : 200) (Molecular Probes, Invitrogen, Eugene, OR, USA). Alexa Fluor 488 phalloidin green fluorescence conjugate (1 : 200, Molecular Probes) and TOPRO (1 : 200, Molecular Probes) have been used to stain cytoskeleton actin and nuclei, respectively. Stained cells were observed at confocal laser scanning microscopy (LSM510META, Zeiss).

### 2.9. RNA Extraction and TaqMan^®^ Quantitative Real-Time PCR

Total RNA was extracted from hPDLSCs and MS-hPDLSCs at P2 and P15 using the RNeasy Mini Kit (Quiagen, Hilden, Germany). 2 *μ*g of RNA from each sample was reverse transcribed using the High Capacity RNA-to-cDNA Kit (Applied Biosystems, Foster, UK). To analyze mesengenic and neurogenic differentiation, appropriate mRNA transcripts (RUNX-2 and ALP for osteogenesis; PPAR*γ* and FABP4 for adipogenesis; ACAN and COL2A1 for chondrogenesis; and Nestin and Enolase for neurogenesis) were evaluated as reported by Rajan et al. [11]. Moreover, quantitative real-time-PCR (qRT-PCR) was performed to assess the expression of genes linked with stemness properties using a 96-well TaqMan Array Human Transcriptional Regulatory Network in an embryonic stem cell kit as per the manufacturer's instructions and run on an Abi 7900HT Sequencing Detection System (Applied Biosystems). Each plate contains 42 assays related to the transcription factors associated with stem cell pluripotency, self-renewal, cell proliferation, and extraembryonic, endodermal, mesodermal, and ectodermal lineage differentiation. The amplification cycle was 10 minutes at 95°C followed by 40 cycles of 15 seconds at 95°C and 1 minute at 60°C. Three independent experiments were run for each condition for a total of 18 plates. Each sample was run as a duplicate in the same plate. Real-time data were analyzed by DataAssist software (Applied Biosystems). A global normalization analysis was used, and GAPDH, 18s, and HPRT1 were chosen as selected internal controls. Only genes showing no outliers replicate, and a maximum cycle threshold (Ct) value = 35 was included in the analysis. A gene was considered differentially expressed when showing a *p* value < 0.05; *p* values were adjusted using Benjamini-Hochberg FDR correction. Ingenuity pathways analysis (IPA) software (Ingenuity Systems, Redwood City, CA, USA) was employed to infer biological functions of the selected gene datasets. IPA predicts functional characterization based on known gene functional interactions and ranks them by a significance score. *p16* and *p21* gene expression was analyzed by qRT-PCR using the same cDNA employed for expression arrays. Specific primer and probe sets were purchased (ThermoFisher Scientific, Waltham, MA, USA; *p16* Hs00923894_m1; *p21* Hs01040810_m1). qRT-PCR was performed in a total volume of 30 *μ*l containing the KAPA Probe Fast Abi Pism qPCR Kit (KAPA Biosystems, 25 ng of cDNA and 1 *μ*l of primer-probe mixture (20x)) on an Abi 7900HT sequencing detection system. The selected genes' relative expression was corrected against endogenous control GAPDH (Hs02758991; ThermoFisher Scientific). Real-time amplification conditions applied were as follows: 10 minutes at 95°C followed by 40 cycles of 15 seconds at 95°C and 1 minute at 60°C. Each sample was run as triplicate. The ΔΔCt method was used to compare relative fold changes between samples and control. *t*-test was used to assess the *p* value, considering data significant when *p* < 0.05.

### 2.10. Statistical Analysis

Data were analyzed using GraphPad Prism 6.0 (GraphPad Software, La Jolla, CA). Statistical analyses were performed with one-way ANOVA test, followed by a Bonferroni post hoc test for multiple comparisons. A *p* value of < 0.05 was considered statistically significant. Data were calculated as mean ± S.D. or SEM.

## 3. Results

### 3.1. Cytofluorimetric Evaluation

**Figure 1:**
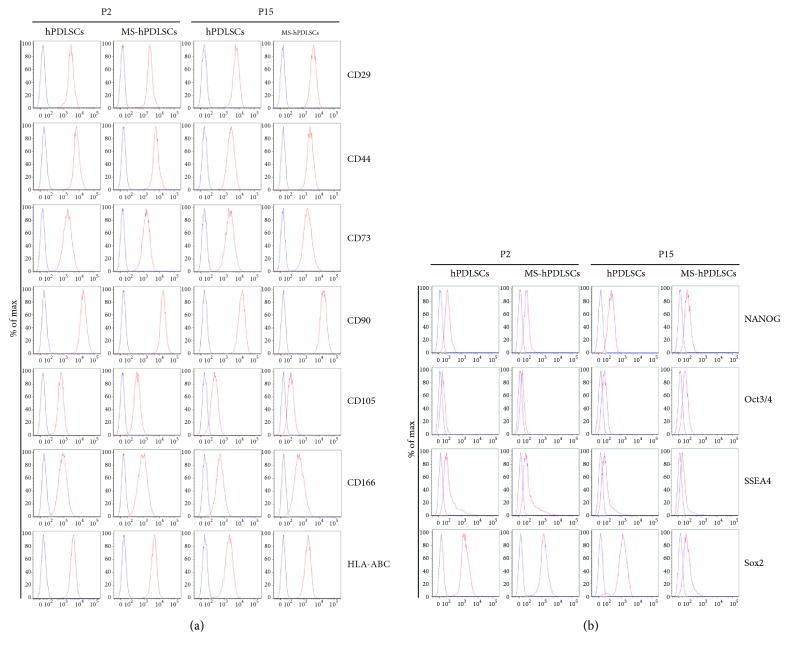
Flow cytometry phenotype of hPDLSCs and MS-hPDLSCs at P2 and P15. (a) Surface antigen expression profile for CD29, CD44, CD73, CD90, CD105, CD166, and HLA-ABC. (b) Intracellular positive marker expression profile for NANOG, SOX2, Oct3/4 and SSEA4. Red histograms show the distribution of each antigen expression, and blue histograms represent the distribution of the respective background control.

Immunophenotypic characterization displayed analogous expression of MSC-associated cell surface markers in hPDLSCs obtained from RR-MS patients and healthy subjects. P2 and P15 hPDLSCs of both healthy donors and RR-MS patients revealed similar phenotype results. Expression of surface molecules such as CD29, CD44, CD73, CD90, CD105, CD166, and human leukocyte antigen- (HLA-) ABC was positive in both donor- and RR-MS patient-derived hPDLSCs at P2 and P15 passages (Figure 1(a)), while that of CD14, CD31, CD34, CD45, CD326, and HLA-DR was negative (data not shown). These results are in parallel with the defining criteria of MSCs [14]. Moreover, pluripotency-associated markers Oct3/4, NANOG, SOX2 and SSEA4 were positive in P2 and P15 hPDLSCs of donors and RR-MS patients (Figure 1(b)).

### 3.2. Proliferation Ability

**Figure 2:**
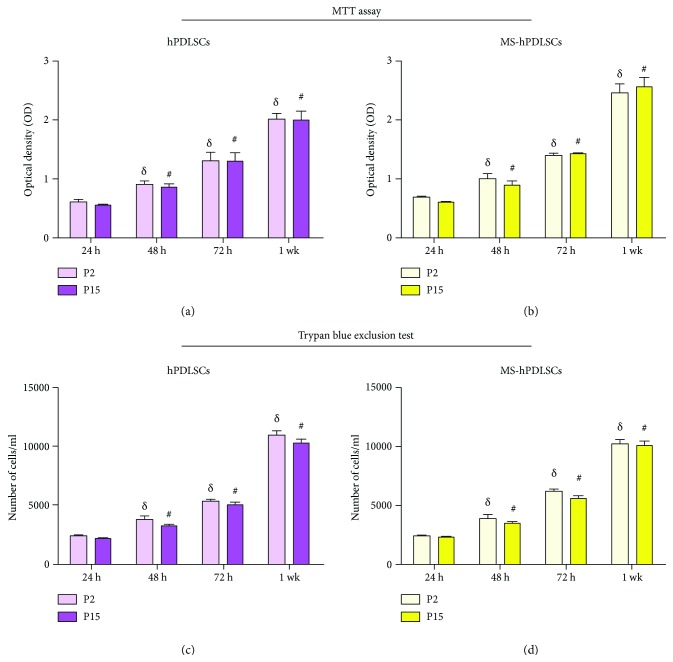
Cell proliferation and viability abilities. Cell proliferation and viability of P2 and P15 hPDLSCs at different time points were evaluated by the 3-(4,5-dimethylthiazol-2-yl)-2,5-diphenyltetrazolium-bromide (MTT) assay and trypan blue exclusion test. Histograms show MTT assay and trypan blue assay data in P12 and P15 of normal hPDLSCs ((a) and (c), resp.) and MS-hPDLSCs ((b) and (d), resp.).Values are expressed as mean ± S.D. *^δ^*Statistically significanct differences for each time for cells at P2. ^#^Statistically significant differences for each time for cells at P15.

Cell proliferation and viability abilities were determined using 3-(4,5-dimethyl-thiazol)-2,5-diphenyl-tetrazolium bromide (MTT) assay and Trypan blue exclusion test. Proliferation rate of hPDLSCs and MS-hPDLSCs was detected at 24, 48, and 72 h and 1 wk of culture. We noticed that both P2 and P15 cells showed a similar gradual increase in their proliferation rates over a one-week incubation period, regardless of whether they were obtained from healthy donors or RR-MS patients (Figures 2(a) and 2(b)). Trypan blue exclusion analysis showed similar viability rate in both P2 and P15 hPDLSCs of healthy donors and RR-MS patient origin (Figures 2(c) and 2(d), resp.).

### 3.3. Mesengenic Differentiation Capacity

**Figure 3:**
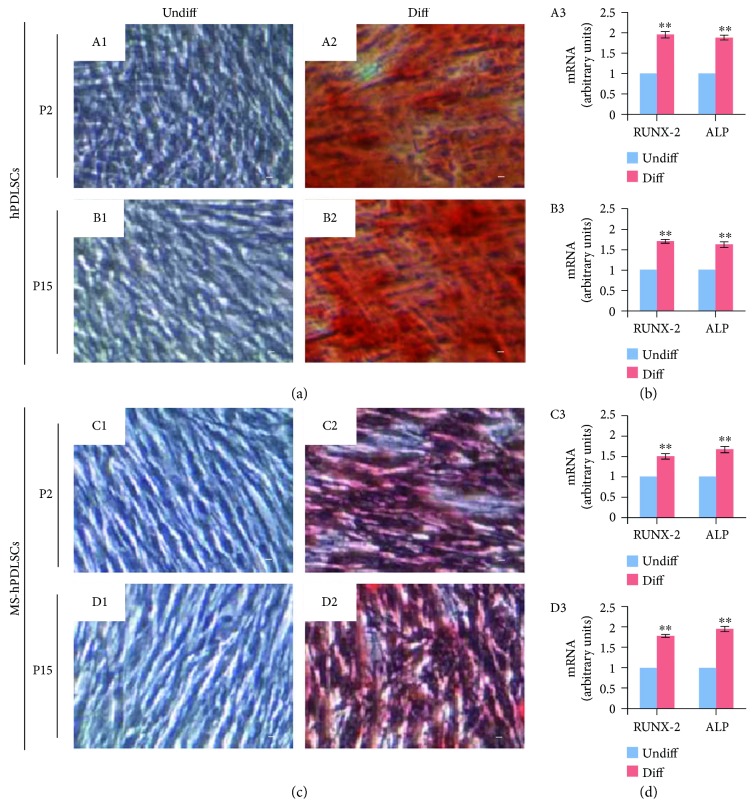
Osteogenic differentiation potential of hPDLSCs collected from healthy donors and MS patients. (a) Photomicrographs of hPDLSCs at P2 (A1) and P15 (B1) under standard conditions and hPDLSCs at P2 (A2) and P15 (B2) at the end of osteogenic commitment. (c) MS-hPDLSCs at P2 (C1) and P15 (D1) under standard conditions and MS-hPDLSCs at P2 (C2) and P15 (D2) at the end of osteogenic differentiation. Differentiation potential was assessed by the formation of calcium-rich hydroxyapatite detected with Alizarin Red staining. Mag: 10x. Bar: 10 *μ*m. Osteogenic differentiation capacity was further confirmed by qRT-PCR for osteogenic markers RUNX-2 and ALP in P2 and P15 of (b) hPDLSCs ((A3) and (B3), resp.) and (d) MS-hPDLSCs ((C3) and (D3), resp.). ^∗∗^*p* < 0.01. Data are expressed as mean ± SEM.

Then, we have investigated the mesengenic differentiation capacities of hPDLSCs. Alizarin red S staining showed positive staining for osteogenic differentiation under osteogenesis-inducing culture conditions. P2 and P15 hPDLSCs of donors (Figure 3(a), A2 and B2, resp.) and MS patients (Figure 3(c), C2 and D2, resp.) showed positivity for Alizarin red S staining. Undifferentiated P2 and P15 hPDLSCs of donors (Figure 3(a), A1 and B1, resp.) and those of MS patients (Figure 3(c), C1 and D1, resp.) were negative for Alizarin red S staining. qRT-PCR results indicated enhanced expression of osteogenic differentiation-associated RUNX-2 and ALP mRNAs in P2 and P15 hPDLSCs of donors (Figure 3(b), A3 and B3, resp.) and in P2 and P15 hPDLSCs of MS patients (Figure 3(c), C3 and D3, resp.).

**Figure 4:**
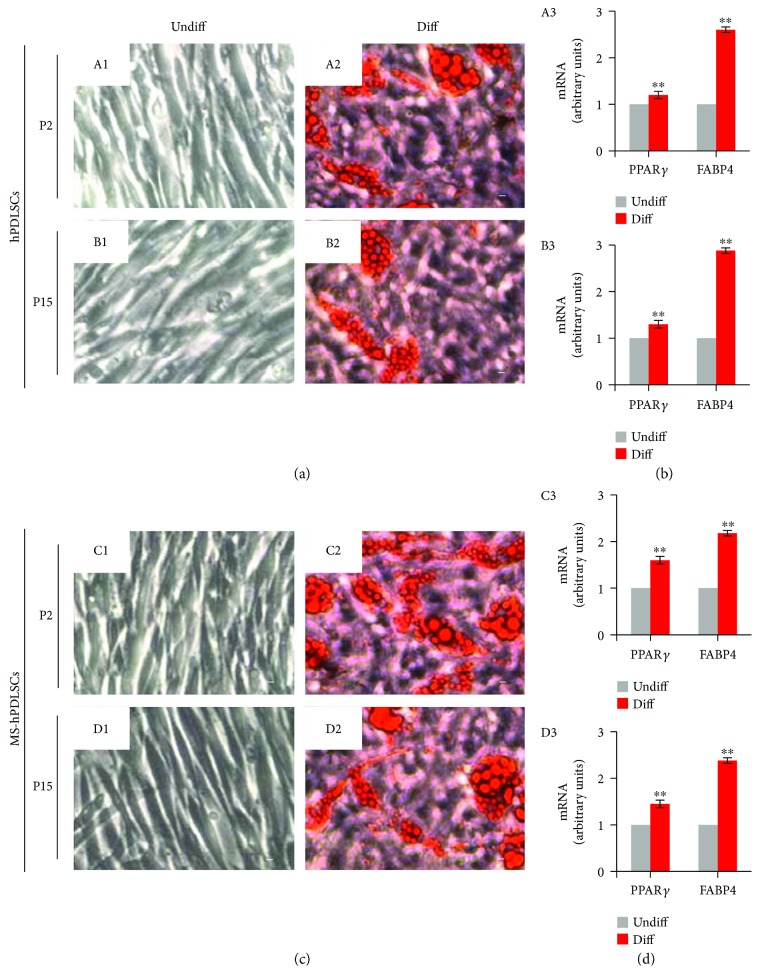
Adipogenic differentiation potential of hPDLSCs collected from healthy donors and from MS patients. (a) Photomicrographs of hPDLSCs at P2 (A1) and P15 (B1) under standard conditions and hPDLSCs at P2 (A2) and P15 (B2) at the end of adipogenic commitment. (c) MS-hPDLSCs at P2 (C1) and P15 (D1) under standard conditions and MS-hPDLSCs at P2 (C2) and P15 (D2) at the end of adipogenic differentiation. Differentiation potential was evaluated by the formation of cytoplasmic lipid droplets and vacuoles (appear as cherry red spheres) detected with Oil Red O staining. Mag: 10x. Bar: 10 *μ*m. Adipogenic differentiation capacity was further confirmed by qRT-PCR for adipogenic markers PPAR*γ* and FABP4 in P2 and P15 of (b) hPDLSCs ((A3) and (B3), resp.) and (d) MS-hPDLSCs ((C3) and (D3), resp.). ^∗∗^*p* < 0.01. Data are expressed as mean ± SEM.

Oil Red O staining showed positive staining for adipogenic differentiation under adipogenesis-inducing culture conditions. P2 and P15 hPDLSCs of donors (Figure 4(a), A2 and B2, resp.) and MS patients (Figure 4(c), C2 and D2, resp.) showed positivity for Oil Red O staining. Undifferentiated P2 and P15 hPDLSCs of donors (Figure 4(a), A1 and B1, resp.) and those of MS patients (Figure 4(c), C1 and D1, resp.) displayed negative staining for Oil Red O staining. qRT-PCR results displayed enhanced expression of adipogenic differentiation-associated PPAR*γ* and FABP4 mRNAs in P2 and P15 hPDLSCs of donors (Figure 4(b), A3 and B3, resp.) and in P2 and P15 hPDLSCs of MS patients (Figure 4(d), C3 and D3, resp.).

**Figure 5:**
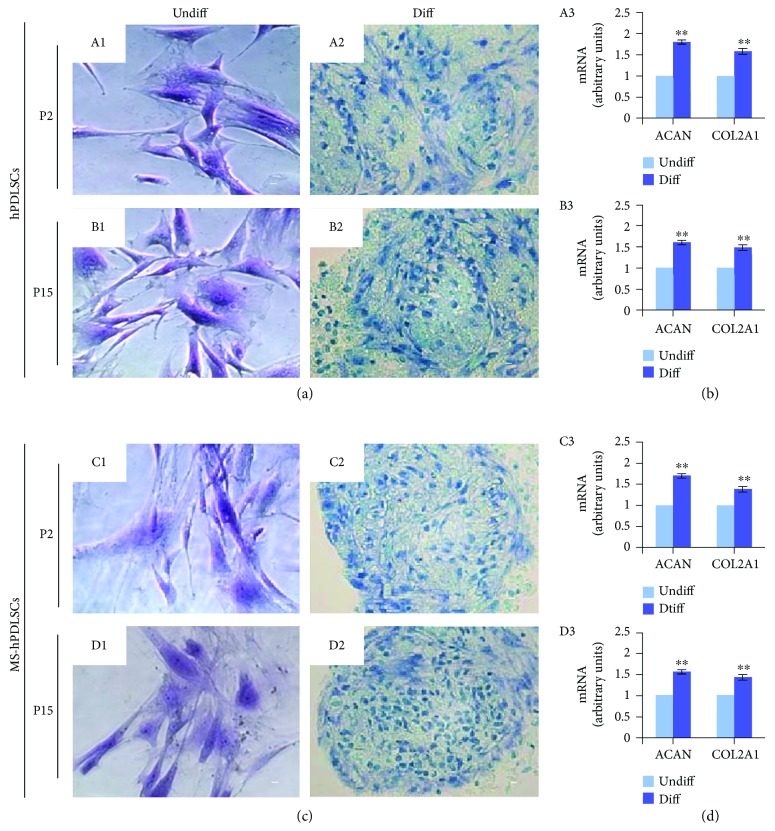
Chondrogenic differentiation potential of hPDLSCs collected from healthy donors and from MS patients. (a) Photomicrographs of hPDLSCs at P2 (A1) and P15 (B1) under standard conditions and hPDLSCs at P2 (A2) and P15 (B2) at the end of chondrogenic commitment. (c) MS-hPDLSCs at P2 (C1) and P15 (D1) under standard conditions and MS-hPDLSCs at P2 (C2) and P15 (D2) at the end of chondrogenic differentiation. Differentiation potential was evaluated by the nodule formation and the positivity to alcian blue staining. Mag: 10x. Bar: 10 *μ*m. Chondrogenic differentiation capacity was confirmed by qRT-PCR for chondrogenic markers ACAN and COL2A1 in P2 and P15 of (b) hPDLSCs ((A3) and (B3), resp.) and (d) MS-hPDLSCs ((C3) and (D3), resp.). ^∗∗^*p* < 0.01. Data are expressed as mean ± SEM.

Alcian blue staining displayed positive staining for chondrogenic differentiation under chondrogenesis culture conditions. P2 and P15 hPDLSCs of donors (Figure 5(a), A2 and B2, resp.) and MS patients (Figure 5(c), C2 and D2, resp.) showed positivity for Alcian blue staining. Undifferentiated P2 and P15 hPDLSCs of donors (Figure 5(a), A1 and B1, resp.) and MS patients hPDLSCs (Figure 5(c), C1 and D1, resp.) revealed negative staining for Alcian blue staining. Chondrogenic markers ACAN and COL2A1 were significantly increased under chondrogenic-inducing conditions in both P2 and P15 hPDLSCs derived from donors (Figure 5(b), A3 and B3, resp.) and MS patients (Figure 5(d), C3 and D3, resp.).

### 3.4. Neurogenic Differentiation Capacity

**Figure 6:**
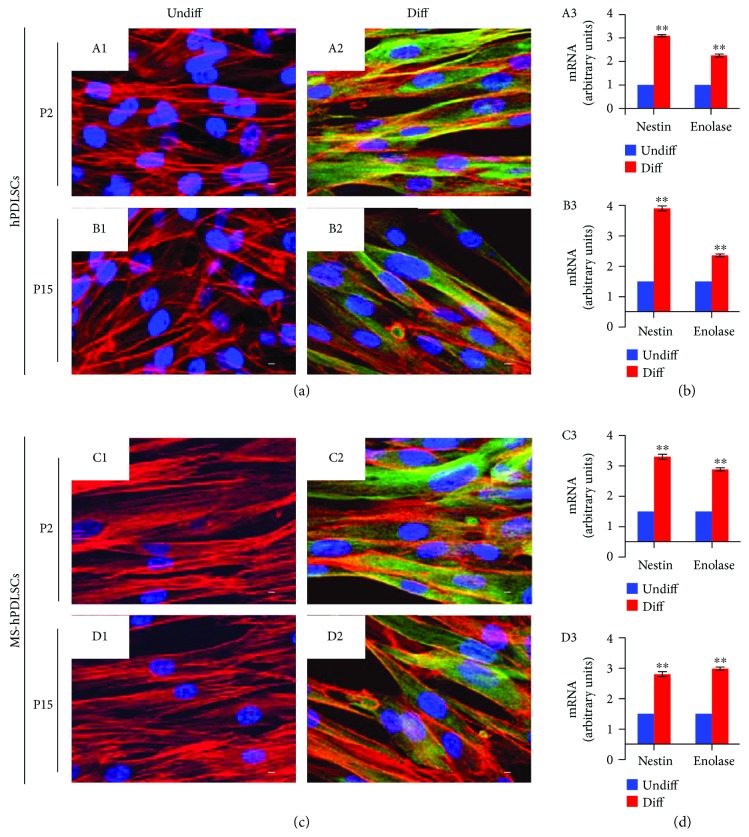
Neurogenic differentiation potential of hPDLSCs collected from healthy donors and from MS patients. (a) hPDLSCs at P2 (A1) and P15 (B1) and neurogenically induced hPDLSCs at P2 (A2) and P15 (B2) passages positive to *β*III-tubulin. (c) MS-hPDLSCs at P2 (C1) and P15 (D1) and neurogenically induced MS-hPDLSCs at P2 (C2) and P15 (D2) passages positive to *β*III-tubulin. All differentiated cells displayed different cytoskeleton morphologies detected with actin rearrangement. Mag: 63x. Bar: 10 *μ*m. Neurogenic differentiation capacity was further confirmed by qRT-PCR for neurogenic markers nestin and enolase in P2 and P15 of (b) hPDLSCs ((A3) and (B3), resp.) and (d) MS-hPDLSCs ((C3) and (D3), resp.). ^∗∗^*p* < 0.01. Data are expressed as mean ± SEM.

In addition to the mesengenic differentiation capacity, we have studied the neurogenic differentiation capacity of hPDLSCs. Under neurogenesis induction, hPDLSCs displayed neuron-like morphological changes. Both P2 and P15 hPDLSCs of donors (Figure 6(a), A2 and B2, resp.) and MS patients (Figure 6(c), C2 and D2, resp.) showed neuron-like cytoskeleton actin rearrangement and positivity to *β*III-tubulin marker. qRT-PCR results revealed enhanced expression of neurogenic differentiation-associated nestin and enolase mRNAs in P2 and P15 hPDLSCs of donors (Figure 6(b), A3 and B3, resp.) and in P2 and P15 hPDLSCs of MS patients (Figure 6(d), C3 and D3, resp.).

### 3.5. Senescence Marker Evaluation

**Figure 7:**
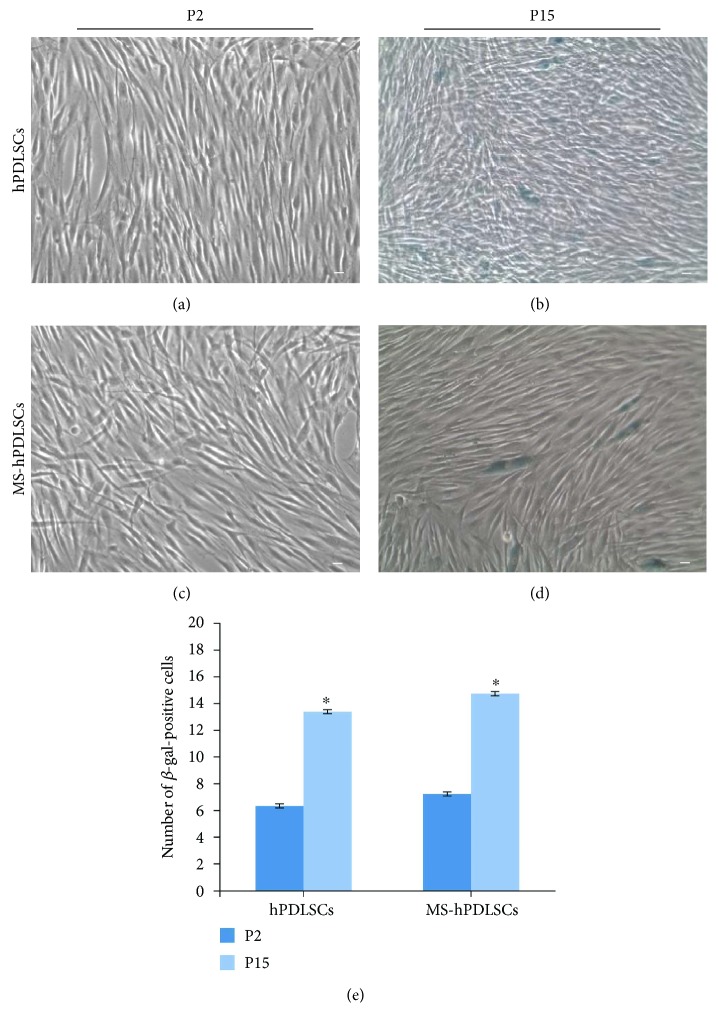
Cellular senescence assessment. hPDLSCs (a) and MS-hPDLSCs (c) at P2 showed a basal staining for X-gal blue solution, while significant positive staining was observed in hPDLSCs (b) and MS-hPDLSCs (d) at P15. Mag:10x. Bar: 10 *μ*m. Histogram shows number of X-gal blue-positive hPDLSCs and MS-hPDLSCs at P2 and P15 (e).^∗^*p* < 0.05. Data are expressed as mean ± SEM.

**Figure 8:**
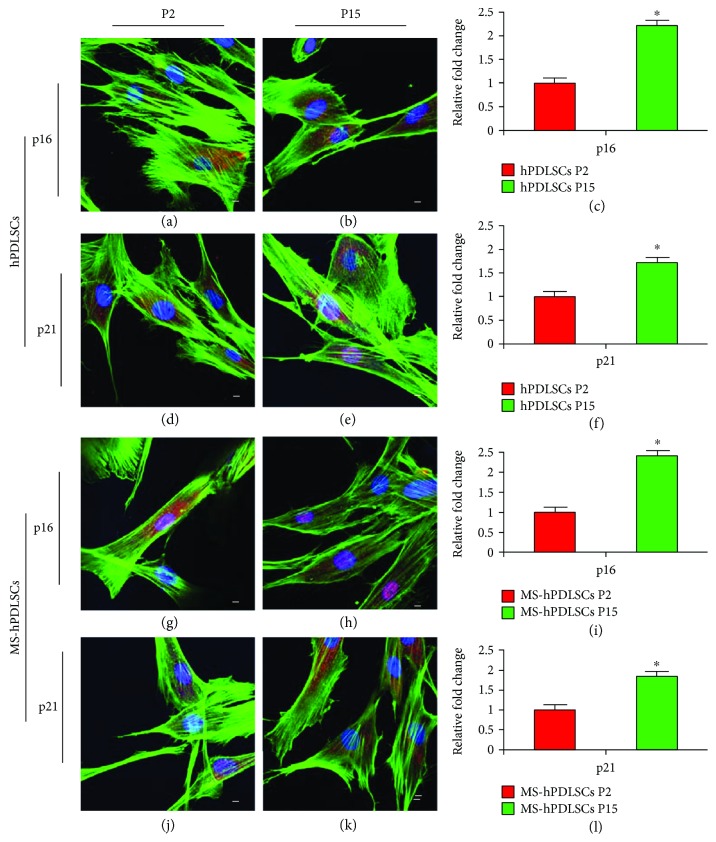
Senescence markers p16 and p21 expression. Immunofluorescence staining of p16 in P2 and P15 hPDLSCs ((a) and (b), resp.) and p21 in P2 and P15 hPDLSCs ((d) and (e), resp.). Histograms show relative fold changes of qRT-PCR for p16 (c) and p21 (f) in hPDLSCs. Immunofluorescence staining of p16 in P2 and P15 MS-hPDLSCs ((g) and (h), resp.) and p21 in P2 and P15 MS-hPDLSCs ((j) and (k), resp.). Histograms show relative fold changes of qRT-PCR for p16 (i) and p21 (l) in MS-hPDLSCs. Green, red, and blue fluorescence was applied to stain actin cytoskeleton, p16 or p21, and nuclei, respectively. Mag: 63x. Bar: 20 *μ*m. ^∗^*p* value < 0.05. Data are expressed as mean ± SEM.

As prolonged passages result in senescence, we have assessed whether hPDLSCs would undergo senescence after extended passages. Senescence-associated X-gal staining showed a mild positive staining for P2 hPDLSCs of donors (Figure 7(a)) and MS patients (Figure 7(c)). On the other hand, a marked positive X-gal staining was observed in P15 hPDLSCs of donors (Figure 7(b)) and MS patients (Figure 7(d)). Immunofluorescence results revealed a minimal positive staining for senescence-linked proteins p16 and p21 in P2 hPDLSCs (Figures 8(a) and 8(d), resp.); however, increased positive staining for p16 and p21 was noticed in P15 hPDLSCs (Figures 8(b) and 8(e), resp.). These results were confirmed with qRT-PCR analysis for p16 and p21 in hPDLSCs (Figures 8(c) and 8(f), resp.). Similar results were observed in hPDLSCs derived from MS patients. Basal level of staining was observed for p16 and p21 in P2 MS-hPDLSCs (Figures 8(g) and 8(j), resp.), while enhanced positive staining was noticed for p16 and p21 in P15 MS-hPDLSCs (Figures 8(h) and 8(k), resp.). qRT-PCR data confirmed the differential expression of p16 and p21 in MS-hPDLSCs (Figures 8(i) and 8(l), resp.).

### 3.6. Gene Expression

**Figure 9:**
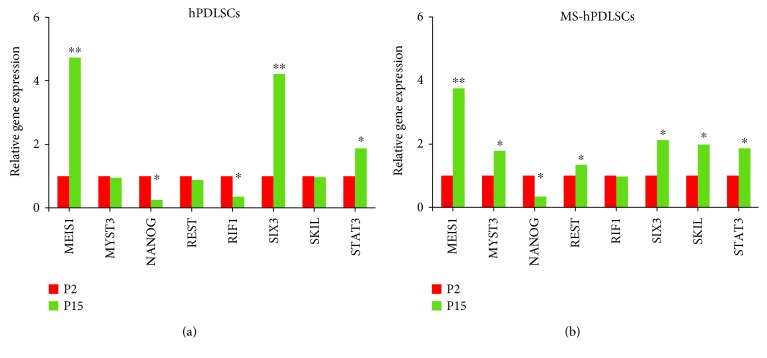
Modulation of genes associated with stemness characteristics at late passage. Histograms show relative expression of mRNA transcripts associated with pluripotency, self-renewal, cell proliferation, and differentiation in hPDLSCs (a) and MS-hPDLSCs (b) at P15. Expression levels of transcripts for P2 and P15 are shown in red and green, respectively. DataAssist software was employed to run a global normalization analysis by using GAPDH, 18s, and HPRT1 as selected internal controls. The reported transcripts evidenced a ^∗^*p* value < 0.05 and ^∗∗^*p* value < 0.01. Data were analyzed using GraphPad Prism 6.0 (GraphPad Software, La Jolla, CA). *p* values were adjusted using Benjamini-Hochberg FDR correction. Data are expressed as mean ± SEM.

**Figure 10:**
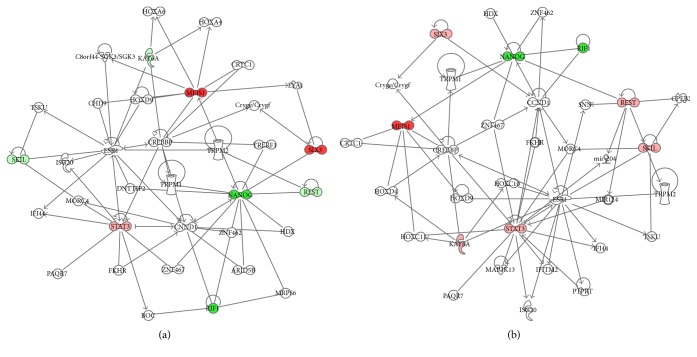
IPA network analysis. Peak scoring networks from NANOG, MEIS1, and SIX3, which regulate stemness properties, in P15 hPDLSCs (a) and P15 MS-hPDLSCs (b) are shown. Upregulated genes are shown in red. Downregulated genes are shown in green. Genes not existing in the LRG list but showed interaction with the LRG list are shown as white open nodes. A solid line depicts a direct functional interaction between the gene products. An arrow depicts functional target of the gene product. LRG: locus reference genomic sequence.

Lastly, we investigated if hPDLSCs and MS-hPDLSCs could display stemness properties including pluripotency and self-renewal abilities after extended passages. Transcriptional regulatory network analysis revealed differential modulation of expression of genes linked with stemness characteristics between early and late passages in both hPDLSCs and MS-hPDLSCs (Figures 9(a) and 9(b), resp.). Pluripotency and sell-renewal marker Meis Homeobox-1 (MEIS1) was increased, while Nanog homeobox (NANOG) was reduced in late passages. Stem cell proliferation marker signal transducer and activator of transcription 3 (STAT3) was enhanced, while replication timing regulatory factor 1 (RIF1) was downregulated. Neural differentiation marker SIX homeobox 3 (SIX3) was upregulated in P15. Other stemness-linked markers lysine acetyltransferase 6A (MYST3), RE1-silencing transcription factor (REST), and SKI-like protooncogene (SKIL) were differentially modulated between hPDLSCs and MS-hPDLSCs. When comparing MS-hPDLSCs with hPDLSCs at both P2 and P15, the modulation of pluripotency and self-renewal marker was found. In particular at P2, MS-hPDLSCs showed the upexpression of NANOG, POU5F1, MEIS1, and SOX2 whereas the expression of SET, SIX3 and SMARCAD1 was reduced compared to hPDLSCs. Interestingly, NEUROG1 showed an increase in relative expression of more than 6 folds (Supplementary Figure 1A). At P15, MS-hPDLSCs maintained an overexpression of NANOG and NEUROG1 together with an evident increase of JARID. SET and SIX3 were confirmed downregulated whereas a recovery in the relative RNA abundance of SMARCAD1 was observed. POU5F1 and SOX2 resulted unmodulated in MS-hPDLSCs at P15 (Supplementary Figure 1B). Differential regulation of these genes was reflected in IPA. Network analysis by IPA showed that in addition to the common pathways, additional distinct pathways were also activated in P15 of hPDLSCs (Figure 10(a)) and MS-hPDLSCs (Figure 10(b)).

## 4. Discussion

Results from clinical trials propose MSC-based treatment as a promising therapeutic tool for RR-MS patients [5, 15]. Among other adult tissues, dental tissues certainly deserve a special attention as an alternative autologous MSC resource due to the requirement of minimal invasive surgical procedure [9]. In the present study, we investigated the potential of hPDLSCs as an effective autologous MSC resource for RR-MS patients. For successful autologous stem cell therapy, it is important that MSCs from the patients must exert normal phenotypic, proliferation, and functional properties, which otherwise would cause crucial impacts in the treatment and recovery. To this end, we performed a comparative analysis on the stemness properties of hPDLSCs derived from RR-MS patients and healthy donors. Given that stem cell therapies might require MSC expansion, it is necessary to examine the stemness properties in prolonged *in vitro* cultures as well. Accordingly, we investigated hPDLSCs at passage 2 and passage 15.

In our study, we found that surface antigen markers CD29, CD44, CD73, CD90, CD105, CD166, and HLA-ABC were positive in healthy donor-derived P2 and P15 hPDLSCs. Interestingly, similar expression of these surface antigens was also found in MS patient-derived P2 and P15 hPDLSCs. These results suggest that MS-linked cellular and biochemical changes did not modulate the stemness properties of hPDLSCs obtained from MS patients at both early and late passages. Moreover, we evaluated cell proliferation and viability abilities. MTT assay and Trypan blue staining assay showed similar cell proliferation and viability capacities in P2 hPDLSCs derived from both MS patients and healthy subjects. Interestingly, we found no defects in the cell viability and cell proliferation abilities of P15 hPDLSCs as well.

Next, we evaluated the differentiation capacity of hPDLSCs towards mesengenic and neurogenic cell lineages. Both MS patients and healthy donor-derived P2 hPDLSCs displayed significant differentiation abilities into osteogenic, adipogenic, chondrogenic, and neurogenic lineages, suggesting stemness characteristics of MS patient-derived hPDLSCs are independent of MS pathology. Previous studies on stemness characteristics of MSCs from patients with inflammatory diseases revealed mixed findings. For example, MSCs obtained from patients with other autoimmune diseases such as rheumatoid arthritis, systemic sclerosis, and systemic lupus erythematosus showed considerable impairments in proliferation and differentiation properties [16–18]. However, in another study, MSCs derived from ALS patients showed similar immunophenotype, proliferation, and differentiation abilities as that of healthy donors [19]. Indeed, our results are in parallel with a previous study in which it has been demonstrated that bone marrow-derived MSCs from secondary progressive MS patients and healthy donors exerted similar proliferation and differentiation capacities [20]. Thus, our results showed that MSC stemness properties are not modulated by MS pathologic microenvironment. In addition, in our study, we showed analogous osteogenic, adipogenic, and neurogenic differentiation capacities in P15 hPDLSCs similar to that of P2 hPDLSCs. These results suggest that hPDLSCs might be a potential choice where it requires extension of MSC passages during stem cell therapy.

In our study, we studied the senescence property of hPDLSCs. We noticed a moderate expression of senescence-associated *β*-galactosidase activity in P15 hPDLSCs of both MS patients and healthy subjects, while P2 cells showed basal level. In addition, senescence markers p16 and p21 were considerably elevated at P15. These results suggested that hPDLSCs might undergo senescence during prolonged *in vitro* passages; however, they sustain their cell proliferation and differentiation properties for longer period under appropriate culture conditions [21]. Additional experiments are required to investigate the senescence property of hPDLSCs during long-term expansion after passage 15 and its effect on proliferation and differentiation properties.

Lastly, we investigated whether prolonged subculture of hPDLSCs and MS-hPDLSCs may affect their stemness properties. We found that genes associated with essential stemness characteristics such as pluripotency, self-renewal, cell proliferation, and differentiation were differentially modulated at late passage. For example, pluripotency and sell-renewal marker MEIS1 was upregulated, while NANOG expression was downregulated at P15 in both normal hPDLSCs and MS-hPDLSCs. Cell proliferation marker STAT3 was upregulated, while another proliferation marker RIF1 was downregulated. SIX3, a neuroectodermal marker, was upregulated during extended passages, suggesting the putative spontaneous differentiation capacity towards neuronal precursor cell lineages. These data suggest that prolonged passages of hPDLSCs may produce modifications in the expression of genes associated with pluripotency, proliferation, and differentiation. Interestingly, we noticed that some gene transcripts were differentially modulated between normal hPDLSCSs and MS-hPDLSCs at late passage. Pluripotency-associated transcripts MYST3, REST, and SKIL were modestly increased in MS-hPDLSCs, while their expression was slightly decreased in normal hPDLSCs in P15. IPA analysis for transcriptional regulatory network suggested that, in addition to the common unidirectionally modulated pathways, some additional but distinct pathways were also activated in normal and in MS-hPDLSCs at extended passages. Given the similar expression of surface antigens and pluripotency markers and no differences in cell proliferation and mesengenic/neurogenic differentiation abilities between normal hPDLSCs and MS-hPDLSCs in both early and late passages, we assume that the differential modulation of gene transcripts that we observed in our study might have resulted from the interaction of heterogeneous pathways which participated in the regulation and maintenance of stemness-related cellular functions.

## 5. Conclusions

In summary, our results demonstrated that hPDLSCs derived from RR-MS patients exert typical stemness characteristics similar to that of hPDLSCs derived from healthy subjects. Cell morphology, immunophenotypic, proliferation, and differentiation properties were analogous. hPDLSCs of extended passages showed senescence activation; however, no changes were noticed with respect to the stemness properties. We propose that hPDLSCs could be a simple and efficient alternative autologous source for customized stem cell therapy in MS patients.

## Supplementary Materials

Supplementary files Modulation of genes associated with stemness characteristics at early and late passages. Histograms show relative expression of mRNA transcripts associated with pluripotency, self-renewal, cell proliferation and differentiation in hPDLSCs and MS-hPDLSCs at P2 (A) and P15 (B). Expression level of transcripts for hPDLSCs and MS-hPDLSCs are shown in green and violet, respectively. DataAssist software was employed to run a global normalization analysis by using GAPDH, 18s and HPRT1 as selected internal controls. The reported transcripts evidenced a *p* value < 0.05 and ^∗∗^*p* value < 0.01; *p* values were adjusted using Benjamini-Hochberg FDR correction. Data are expressed as mean ± SEM.
