# Supplementary material for: Stemness Characteristics of Periodontal Ligament Stem Cells from Donors and Multiple Sclerosis Patients: A Comparative Study
Source: Stem Cells Int. 2017 Dec 14;2017:1606125. doi: 10.1155/2017/1606125 (PMC5745749; doi:10.1155/2017/1606125)
Supplement: Supplementary files — Modulation of genes associated with stemness characteristics at early and late passages. Histograms show relative expression of mRNA transcripts associated with pluripotency, self-renewal, cell proliferation and differentiation in hPDLSCs and MS-hPDLSCs at P2 (A) and P15 (B). Expression level of transcripts for hPDLSCs and MS-hPDLSCs are shown in green and violet, respectively. DataAssist software was employed to run a global normalization analysis by using GAPDH, 18s and HPRT1 as selected internal controls. The reported transcripts evidenced a p value < 0.05 and ∗∗p value < 0.01; p values were adjusted using Benjamini-Hochberg FDR correction. Data are expressed as mean ± SEM. [file 1606125.f1.pptx]

## Slide 1
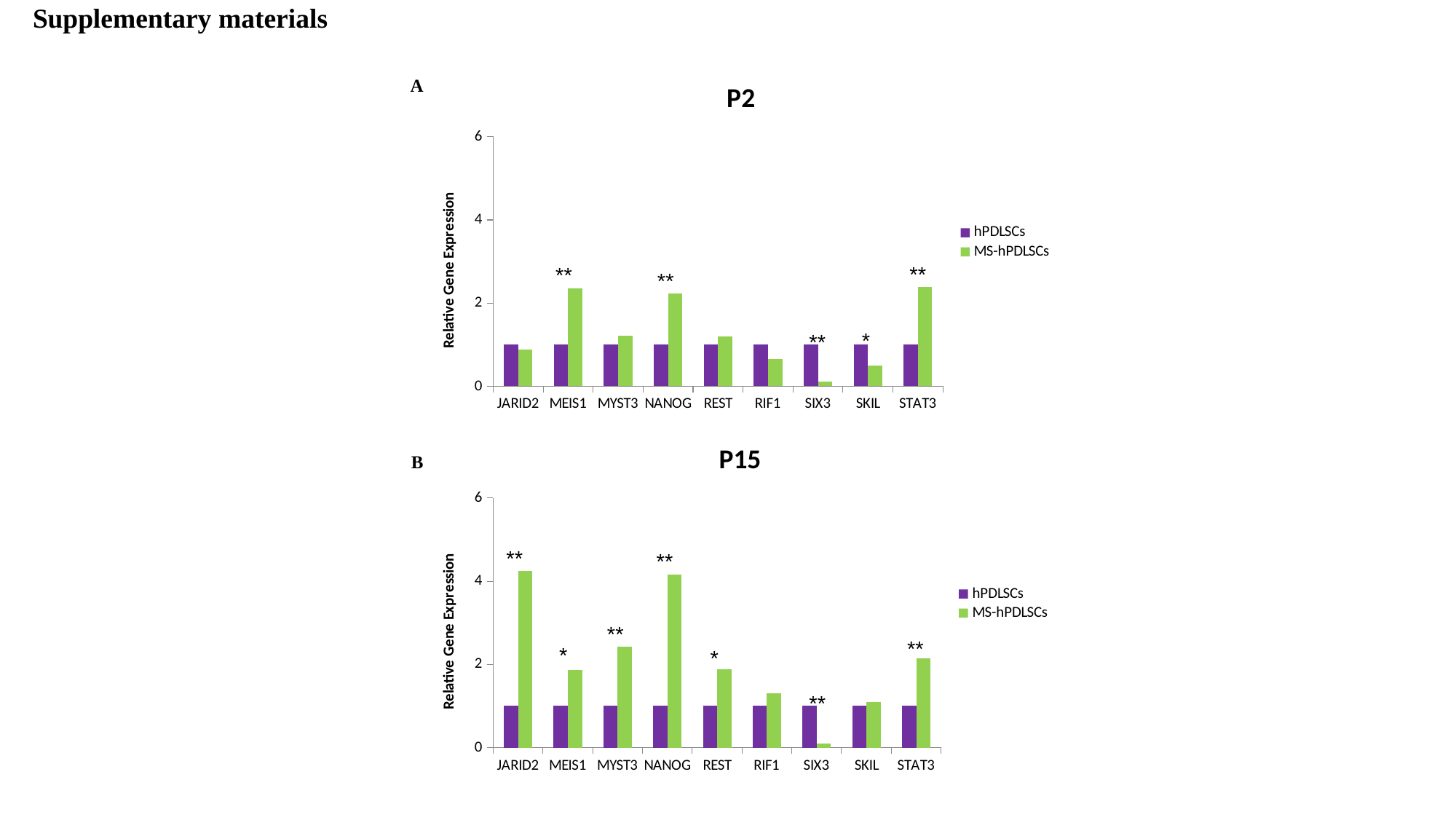

Supplementary materials
### Chart: P2
| Category | hPDLSCs | MS-hPDLSCs |
|---|---|---|
| JARID2 | 1.0 | 0.8900000000000001 |
| MEIS1 | 1.0 | 2.349999999999999 |
| MYST3 | 1.0 | 1.22 |
| NANOG | 1.0 | 2.24 |
| REST | 1.0 | 1.1900000000000004 |
| RIF1 | 1.0 | 0.6500000000000002 |
| SIX3 | 1.0 | 0.12000000000000002 |
| SKIL | 1.0 | 0.5 |
| STAT3 | 1.0 | 2.3899999999999997 |A
**
**
**
*
**
### Chart: P15
| Category | hPDLSCs | MS-hPDLSCs |
|---|---|---|
| JARID2 | 1.0 | 4.25 |
| MEIS1 | 1.0 | 1.87 |
| MYST3 | 1.0 | 2.42 |
| NANOG | 1.0 | 4.159999999999998 |
| REST | 1.0 | 1.8800000000000001 |
| RIF1 | 1.0 | 1.3 |
| SIX3 | 1.0 | 0.09000000000000002 |
| SKIL | 1.0 | 1.1 |
| STAT3 | 1.0 | 2.15 |B
**
**
**
**
*
*
**
